# Two-dimensional coronene fractals: modified reverse degree indices, comparative analysis of information entropy and predictive modeling of spectral properties

**DOI:** 10.3389/fchem.2025.1588942

**Published:** 2025-05-06

**Authors:** A. R. Abul Kalaam, A. Berin Greeni

**Affiliations:** School of Advanced Sciences, Vellore Institute of Technology, Chennai, India

**Keywords:** coronene fractals, modified reverse degree-based indices, information entropy, spectral properties, predictive models

## Abstract

Topological characterization through graph-theoretical methods translates chemical and structural data into quantitative values that represent the molecular system. Our research explores the use of topological indices to study fractal structures. Molecular fractals are complex geometric configurations that exhibit self-similarity at different levels and systematically formed by repeating a fundamental unit. This study focuses on coronene-based molecular fractals, where coronene, a benzenoid molecule with a symmetrical graphite-like structure, finds applications in organic semiconductors, sensors, and molecular electronics, due to its unique electronic and optical properties. Additionally, information entropy is employed to evaluate and compare the structural complexities of coronene fractals. Spectra-based energetic properties such as total π -electron energy, HOMO-LUMO energy gaps, spectral diameter, delocalization and resonance energies are calculated to assess their kinetic and thermodynamic stability. Furthermore, predictive models are provided for estimating spectral characteristics across higher-dimensional coronene fractal structures.

## 1 Introduction

Benzenoid hydrocarbons are a group of polycyclic compounds consisting of six-member linked rings, characterized by their aroma and unique physicochemical properties. These substances create powerful inter-molecular bonds by acting in single and double bonds alternatively (Hill et al., 2004). Higher-order structural co-ordination is indicated by larger π -conjugated complexes. These characteristics primarily make them useful for applications in opto-electronic devices, nanomaterials, and natural semiconductors (Pisula et al., 2010; Pisula et al., 2011). Coronene, a planar molecule with seven peri-fused benzene rings, is well-known for having delocalized π -electrons, extended conjugation, and extreme symmetry (Newman, 1940; Robertson and White, 1945; Popov and Boldyrev, 2012). It serves as a fundamental polycyclic aromatic hydrocarbon (PAH) model for studying larger PAHs, graphene quantum dots, and graphene nanoflakes. Coronene-based structures enable precise theoretical investigations and bridge PAHs with graphene materials (Santa Daría et al., 2024; Tachikawa and Lund, 2022). It has a well-defined structure, fluorescence, and electronic properties which makes it a benchmark in theoretical and experimental studies. Coronene fractals exhibit exceptional electronic, optical, and energy-related properties, with strong π -electron delocalization enhancing charge transport and stable π -conjugation improving the performance of capacitors and batteries (Demir and Üngördü, 2023; Sanyal et al., 2013; Dobrowolski et al., 2011). Molecular stacking and employers are further enhanced by its symmetrical and planar architecture (Fedotov et al., 2013).

Fractal geometry, which explores recurring patterns at different scales, has evolved from describing physical theories to serve various applications such as complexes in medical and molecular engineering, neural networks, and laptop graphics, etc (Kirkby, 1983). Extensive research has been carried out using fractal methods. These deterministic fractals arise by combining benzene with hierarchical structure sequences, making them a significant tool for advancing nanotechnology and biotechnology (Uahabi and Atounti, 2015). Clar aromatic sextet theory is a concept introduced by Erich Clar to describe the electronic structure of polycyclic aromatic hydrocarbons (PAHs). It is particularly useful for understanding resonance, stability, and reactivity in PAH systems (Hosoya, 2005). Fractal molecular architecture, often analyzed through Clar’s system and golden ratio measurements, exhibits scaling properties that demonstrate its adaptability and potential (Lee and Chang, 1996). Studies of coronene-based fractals have shown that they can serve as supports for advanced nanomaterials (Nisha and Senthil Kumar, 2020). Despite significant advances in theoretical research, the integration of these complex systems remains a challenge, requiring further research (Kumar et al., 2017). Recent work emphasis on the unique aromatic properties and scaling behavior of fractal benzenoids, emphasizes their importance in development and fabrication of high-performance nanomaterials for optical and electronic device applications (Duan et al., 2021).

In computational chemistry, topological indices considered as are important tools that provide information on the chemical and structural characteristics of molecules (Estrada and Uriarte, 2001; Kumar and Das, 2024). Among these, the degree-based Zagreb index and the distance-based Wiener index have been crucial in forecasting molecular characteristics, including stability and boiling points (Wiener, 1947; Gutman and Trinajstić, 1972). In this article, we utilize modified reverse degree-based indices that incorporate a variable parameter, “ k ,” which potentially alters the graph’s degree sequence. Unlike traditional methods with fixed-degree sequences, this approach allows customization of the “ k ,” value to better correlate with specific datasets and their properties. This method is not limited to specific indices and can be applied to all degree-based indices. Notably, as the “ k ,” value increases, these modified indices exhibit a high correlation with the physicochemical characteristics of corona, blood cancer, and heart disease treatment drug molecules (Arockiaraj et al., 2023a; Arockiaraj et al., 2023b; Arockiaraj et al., 2024). In addition, they are used for stability analysis in advanced materials like carbon nanosheets, metal-organic frameworks, and pent-heptagonal nanostructures (Abul Kalaam and Berin Greeni, 2024). Further, employing hybrid models allows for more precise predictions of molecular activity (Arockiaraj et al., 2023c).

Entropy analysis is a fundamental method in the field of information theory, which offers special insights into the complexity and stability of molecules. Shannon’s entropy measures structural randomness (Dehmer, 2008; Shannon, 1948), while graph entropy is related to the vertices and edges of molecular graphs, which makes it easier to analyze a system using graph structures. Higher entropy of a structure constitutes more disorderness in the macrostructure, which reduces structural stability. However, high entropy materials, such as high-entropy alloys (HEAs), exhibit unique properties due to their high configurational entropy, which can result in the formation of stable disordered solid solutions. While high entropy promotes disorderness, it can also contribute to distinctive structural stabilities and desirable properties. For instance, HEAs are known for their high strength, ductility, and resistance to wear and corrosion.

Research articles focused on molecular fractals have explored various structural and topological aspects (Malik et al., 2023; Xu and Liu, 2025; Yogalakshmi and Easwaramoorthy, 2024). Recent studies on coronene fractals have examined degree and degree-sum properties, reverse degree-based indices, and coronene frameworks, as discussed in (Arockiaraj et al., 2022; Ullah et al., 2024; Khabyah et al., 2023). This study explores coronene fractal structures, analyzing their entropy levels and complexity through modified reverse degree-based indices. By delving into their structural and spectral features, it aims to deepen our understanding of their stability, complexity, and overall properties.

## 2 Methodology

In this study, we examine three configurations of coronene fractals modeled as two-dimensional molecular graph structure and is represented by G , with |V(G)| and |E(G)| denote the number of vertices and edges, respectively. The degree of a vertex a∈V(G) denoted as d(a) , indicates the number of vertices directly connected to a . The maximum degree, Δ(G) , represents the highest connectivity among all vertices in the graph G . Recent modification in reverse degree is done by introducing a parameter k (with k≥1 ) that enhance the graph degree sequence to closely predict properties (Arockiaraj et al., 2023a). The modified reverse degree, represented $M_{k}Rd\left(a\right)$ , is defined as follows:

$$M_{k}R\left(d(a)\right)=\left\{\begin{array}{cc} \Delta \left(G\right)-d(a)+k\; & :\;k\leq d(a)\\ \Delta \left(G\right)-d(a)+k\left(mod\;\Delta \left(G\right)\right)\; & :\;k>d(a) \end{array}\right.$$

Modified reverse degree-based topological indices, $M_{k}RTI$ , are employed to characterize coronene fractals by evaluating atom connectivity and providing insights into their molecular structure. For a graph G , the formulation of $M_{k}RTI$ is given as:

$$M_{k}RTI\left(G\right)=\underset{ab\in E\left(G\right)}{\sum }M_{k}RTI\left(d\left(a\right),d\left(b\right)\right)=\underset{ab\in E\left(G\right)}{\sum }TI\left(M_{k}R\left(d\left(a\right)\right),M_{k}R\left(d\left(b\right)\right)\right),$$

where ab represents the edge connecting vertices a and b . This formula provides an intensive evaluation by using the contributions of all edges inside the graph. The edge set E(G) is divided into equivalent subsets, such that $E\left(G\right)={⋃}_{i=1}^{n}E_{i}$ . Each subset of $E_{i}$ , where $ab\in E_{i}$ and i=1,2,…,n , groups edges based on vertex connectivity in G . For any subset $E_{i}$ , the corresponding $M_{k}RTI$ is calculated as:

$$M_{k}RTI\left(E_{i}\right)=|E_{i}|\times TI\left(M_{k}R\left(d\left(a\right)\right),M_{k}R\left(d\left(b\right)\right)\right),$$

where $\left|E_{i}\right|$ represents the number of edges in subset $E_{i}$ , and $TI\left(M_{k}R\left(d\left(a\right)\right),M_{k}R\left(d\left(b\right)\right)\right)$ evaluates the contribution of modified reverse degree for the connected vertices.

The total $M_{k}RTI$ for graph G is obtained by summing the contributions from all subsets $E_{i}$ :

$$M_{k}RTI\left(G\right)=\overset{n}{\underset{i=1}{\sum }}|E_{i}|\times TI\left(M_{k}R\left(d\left(a\right)\right),M_{k}R\left(d\left(b\right)\right)\right)$$

The topological index functions based on the modified reverse degree are outlined below.

• Modified reverse first Zagreb index $\left(M_{k}RM_{1}\right)$ :
$$M_{k}RM_{1}\left(d\left(a\right),d\left(b\right)\right)=M_{k}R\left(d\left(a\right)\right)+M_{k}R\left(d\left(b\right)\right)$$ (1)
• Modified reverse second Zagreb index $\left(M_{k}RM_{2}\right)$ :
$$M_{k}RM_{2}\left(d\left(a\right),d\left(b\right)\right)=M_{k}R\left(d\left(a\right)\right)\times M_{k}R\left(d\left(b\right)\right)$$ (2)
• Modified reverse forgotten index $\left(M_{k}RF\right)$ :
$$M_{k}RF\left(d\left(a\right),d\left(b\right)\right)={\left(M_{k}R\left(d\left(a\right)\right)\right)}^{2}+{\left(M_{k}R\left(d\left(b\right)\right)\right)}^{2}$$ (3)
• Modified reverse Sombor index $\left(M_{k}RS\right)$ :
$$M_{k}RS\left(d\left(a\right),d\left(b\right)\right)=\sqrt{{\left(M_{k}R\left(d\left(a\right)\right)\right)}^{2}+{\left(M_{k}R\left(d\left(b\right)\right)\right)}^{2}}$$ (4)
• Modified reverse geometric arithmetic index $\left(M_{k}RGA\right)$ :
$$M_{k}RGA\left(d\left(a\right),d\left(b\right)\right)=\frac{2\cdot \sqrt{M_{k}R\left(d\left(a\right)\right)\times M_{k}R\left(d\left(b\right)\right)}}{M_{k}R\left(d\left(a\right)\right)+M_{k}R\left(d\left(b\right)\right)}$$ (5)
• Modified reverse hyper-Zagreb index $\left(M_{k}RHZ\right)$ :
$$M_{k}RHZ\left(d\left(a\right),d\left(b\right)\right)={\left(M_{k}R\left(d\left(a\right)\right)+M_{k}R\left(d\left(b\right)\right)\right)}^{2}$$ (6)
• Modified reverse harmonic index $\left(M_{k}RH\right)$ :
$$M_{k}RH\left(d\left(a\right),d\left(b\right)\right)=\frac{2}{M_{k}R\left(d\left(a\right)\right)+M_{k}R\left(d\left(b\right)\right)}$$ (7)
• Modified reverse first redefined Zagreb index $\left(M_{k}RReZ_{1}\right)$ :
$$M_{k}RReZ_{1}\left(d\left(a\right),d\left(b\right)\right)=\frac{M_{k}R\left(d\left(a\right)\right)+M_{k}R\left(d\left(b\right)\right)}{M_{k}R\left(d\left(a\right)\right)\times M_{k}R\left(d\left(b\right)\right)}$$ (8)
• Modified reverse second redefined Zagreb index $\left(M_{k}RReZ_{2}\right)$ :
$$M_{k}RReZ_{2}\left(d\left(a\right),d\left(b\right)\right)=\frac{M_{k}R\left(d\left(a\right)\right)\times M_{k}R\left(d\left(b\right)\right)}{M_{k}R\left(d\left(a\right)\right)+M_{k}R\left(d\left(b\right)\right)}$$ (9)
• Modified reverse bi-Zagreb index $\left(M_{k}RBM\right)$ :
$$M_{k}RBM\left(d\left(a\right),d\left(b\right)\right)=M_{k}R\left(d\left(a\right)\right)+M_{k}R\left(d\left(b\right)\right)+\left(M_{k}R\left(d\left(a\right)\right)\times M_{k}R\left(d\left(b\right)\right)\right)$$ (10)
• Modified reverse tri-Zagreb index $\left(M_{k}RTM\right)$ :
$$M_{k}RTM\left(d\left(a\right),d\left(b\right)\right)={\left(M_{k}R\left(d\left(a\right)\right)\right)}^{2}+{\left(M_{k}R\left(d\left(b\right)\right)\right)}^{2}+\left(M_{k}R\left(d\left(a\right)\right)\times M_{k}R\left(d\left(b\right)\right)\right)$$ (11)
• Modified reverse geometric bi-Zagreb index $\left(M_{k}RGBM\right)$ :
$$M_{k}RGBM\left(d\left(a\right),d\left(b\right)\right)=\frac{\sqrt{M_{k}R\left(d\left(a\right)\right)\times M_{k}R\left(d\left(b\right)\right)}}{M_{k}R\left(d\left(a\right)\right)+M_{k}R\left(d\left(b\right)\right)+M_{k}R\left(d\left(a\right)\right)\times M_{k}R\left(d\left(b\right)\right)}$$ (12)

## 3 Evaluation of modified reverse degree indices

**FIGURE 1:**
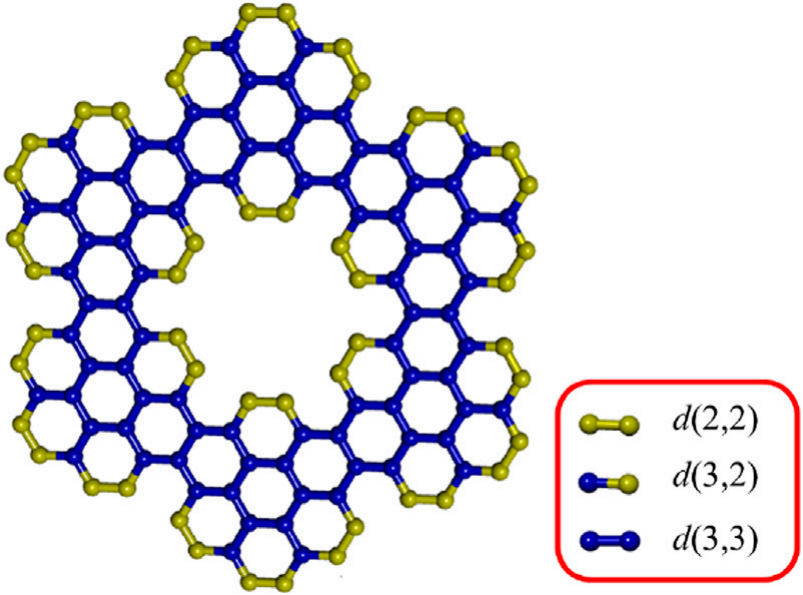
The degree based enumeration of coronene fractal structure.

**FIGURE 2:**
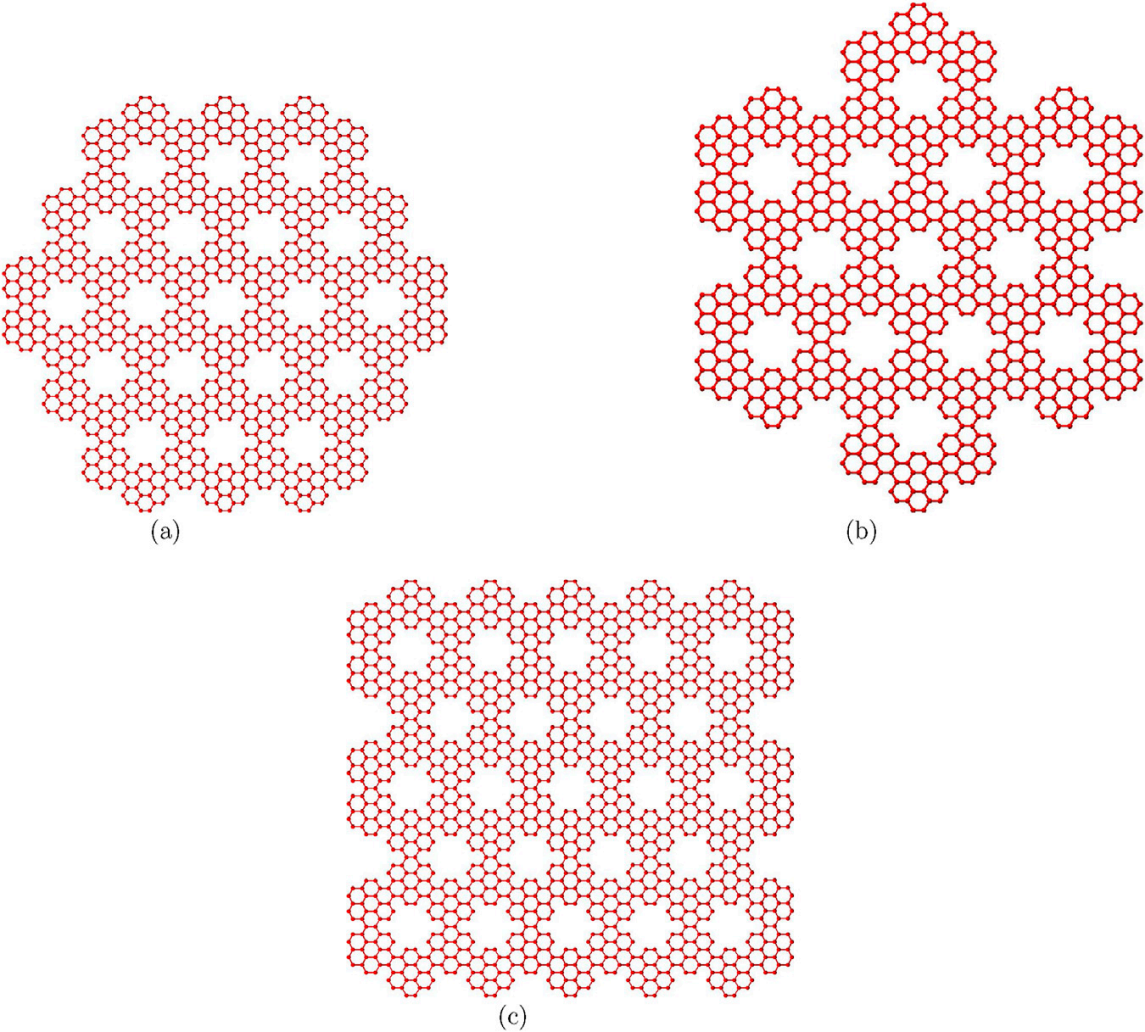
Configurations of coronene fractals **(a)** ZHCF(3) **(b)** AHCF(2) **(c)** RCF(5,3).

We explore three coronene fractal configurations: ZHCF (n) , AHCF (n) , and RCF (m,n) , as illustrated in Figures 1, 2. The structural parameters for these configurations are given by: $|V\left(\text{ZHCF}\left(n\right)\right)|=126n^{2}+6n$ and $|E\left(\text{ZHCF}\left(n\right)\right)|=171n^{2}+3n$ ; for AHCF, these are $|V\left(\text{AHCF}\left(n\right)\right)|=378n^{2}-366n+120$ and $|E\left(\text{AHCF}\left(n\right)\right)|=513n^{2}-507n+168$ ; and for RCF, they are |V(RCF(m,n))|=84mn+2m+46n and |E(RCF(m,n))|=114mn+m+59n . All configurations share a maximum vertex degree of 3. The modified reverse degree metrics for each vertex are as follows:

$$M_{1}R\left(d\left(a\right)\right)=\left\{\begin{array}{cc} 2\; & :\;d\left(a\right)=2\\ 1\; & :\;d\left(a\right)=3 \end{array}\right.$$

$$M_{2}R\left(d\left(a\right)\right)=\left\{\begin{array}{cc} 3\; & :\;d\left(a\right)=2\\ 2\; & :\;d\left(a\right)=3 \end{array}\right.$$

$$M_{3}R\left(d\left(a\right)\right)=\left\{\begin{array}{cc} 1\; & :\;d\left(a\right)=2\\ 3\; & :\;d\left(a\right)=3 \end{array}\right.$$

To calculate the modified reverse topological indices, edge partitioning is used, as illustrated in Figure 1, for three configurations of coronene fractals based on their standard vertex degrees, as detailed in Table 1. Each index involves complex computations with varying parameters. For instance, the calculation of the first Zagreb-based index is demonstrated using ZHCF coronene structures for different values of k=1,2,3 . When the variable parameter is set to k=1 , the degree pairs (2,2), (2,3), and (3,3) are modified to (2,2), (2,1), and (1,1), respectively. Therefore,

$$\begin{array}{ll} M_{1}RM_{1}\left(ZHCF\left(n\right)\right) & =|E\left(2,2\right)|\times M_{1}R\;d\left(2,2\right)+|E\left(2,3\right)|\times M_{1}R\;d\left(2,3\right)+|E\left(3,3\right)|\times M_{1}R\;d\left(3,3\right)\\ & =\left(18n^{2}+6n\right)\times \left(2+2\right)+\left(36n^{2}+12n\right)\times \left(2+1\right)+\left(117n^{2}-15n\right)\times \left(1+1\right)\\ & =414n^{2}+30n \end{array}$$

**TABLE 1:** Degree based edge partition of three configurations of coronene fractals.

Bond type	ab		Coronene fractals		
	d(a)	d(b)	ZHCF(n)	AHCF(n)	RCF(m,n)
C-C	2	2	$18n^{2}+6n$	$54n^{2}-42n+12$	12mn+2m+10n
C-C	2	3	$36n^{2}+12n$	$108n^{2}-84n+24$	24mn+4m+20n
C-C	3	3	$117n^{2}-15n$	$351n^{2}-381n+132$	78mn−5m+29n

For k=2 the degree classes are modified into (3,3), (3,2), and (2,2). Therefore,

$$\begin{array}{ll} M_{2}RM_{1}\left(ZHCF\left(n\right)\right) & =|E\left(2,2\right)|\times M_{1}R\;d\left(2,2\right)+|E\left(2,3\right)|\times M_{1}R\;d\left(2,3\right)+|E\left(3,3\right)|\times M_{1}R\;d\left(3,3\right)\\ & =\left(18n^{2}+6n\right)\times \left(3+3\right)+\left(36n^{2}+12n\right)\times \left(3+2\right)+\left(117n^{2}-15n\right)\times \left(2+2\right)\\ & =756n^{2}+36n \end{array}$$

Similarly for k=3 the degree classes are modified into (1,1), (1,3), and (3,3). Therefore,

$$\begin{array}{ll} M_{3}RM_{1}\left(ZHCF\left(n\right)\right) & =|E\left(2,2\right)|\times M_{1}R\;d\left(2,2\right)+|E\left(2,3\right)|\times M_{1}R\;d\left(2,3\right)+|E\left(3,3\right)|\times M_{1}R\;d\left(3,3\right)\\ & =\left(18n^{2}+6n\right)\times \left(1+1\right)+\left(36n^{2}+12n\right)\times \left(1+3\right)+\left(117n^{2}-15n\right)\times \left(3+3\right)\\ & =882n^{2}+30n \end{array}$$

The modified reverse degree-based indices illustrated in Equations 1–12, combined with the edge partitioning present in Table 1, are employed to compute the $M_{k}RTI$ for three configurations of coronene fractals. The results, corresponding to the variable parameters k=1,2 , and 3, are summarized in Tables 2–4.

**TABLE 2:** Modified reverse degree indices of ZHCF structure for variable parameters k = 1, 2, and 3.

Zigzag hexagonal coronene fractal structure			
$M_{k}RTI$	k=1	k=2	k=3
$M_{k}RM_{1}$	$414n^{2}+30n$	$756n^{2}+36n$	$882n^{2}-30n$
$M_{k}RM_{2}$	$261n^{2}+33n$	$846n^{2}+66n$	$1179n^{2}-93n$
$M_{k}RF$	$558n^{2}+78n$	$1728n^{2}+144n$	$2502n^{2}-138n$
$M_{k}RS$	$296.8731n^{2}+22.5903n$	$537.0936n^{2}+26.2956n$	$635.6868n^{2}-17.2071n$
$M_{k}RGA$	$168.9411n^{2}+2.3137n$	$297.5400n^{2}-8.8210n$	$166.1769n^{2}+1.3923n$
$M_{k}RReZ_{1}$	$306n^{2}-6n$	$159n^{2}-n$	$162n^{2}+18n$
$M_{k}RReZ_{2}$	$100.5n^{2}+6.5n$	$187.2n^{2}+8.4n$	$211.5n^{2}-10.5n$
$M_{k}RH$	$150n^{2}-4n$	$78.9n^{2}-0.7n$	$75n^{2}+7n$
$M_{k}RHZ$	$1080n^{2}+144n$	$3420n^{2}+276n$	$4860n^{2}-324n$
$M_{k}RBM$	$675n^{2}+63n$	$1602n^{2}+102$	$2061n^{2}-123n$
$M_{k}RTM$	$819n^{2}+111n$	$2574n^{2}+210n$	$3681n^{2}-231n$
$M_{k}RGBM$	$53.6823n^{2}-0.1059n$	$40.8668n^{2}+0.1222n$	$38.3077n^{2}+1.9692n$

**TABLE 3:** Modified reverse degree indices of AHCF structure for variable parameters k = 1, 2, and 3.

Armchair hexagonal coronene fractal structures			
$M_{k}RTI$	k=1	k=2	k=3
$M_{k}RM_{1}$	$1242n^{2}-1182n+384$	$2268n^{2}-2196n+720$	$2646n^{2}-2706n+912$
$M_{k}RM_{2}$	$783n^{2}-717n+228$	$2538n^{2}-2406n+780$	$3537n^{2}-3723n+1272$
$M_{k}RF$	$1674n^{2}-1518n+480$	$5184n^{2}-4896n+1584$	$7506n^{2}-7782n+2640$
$M_{k}RS$	$890.6194n^{2}-845.4390n+274.2829$	$1611.3n^{2}-1558.7n+510.7973$	$1907.1n^{2}-1941.5n+652.8938$
$M_{k}RGA$	$506.8224n^{2}-502.1952n+166.6272$	$892.6092n^{2}-910.2516n+306.3576$	$498.5280n^{2}-495.7440n+164.7840$
$M_{k}RReZ_{1}$	$918n^{2}-930n+312$	$477n^{2}-479n+160$	$486n^{2}-450n+144$
$M_{k}RReZ_{2}$	$301.5n^{2}-288.5n+94$	$561.6n^{2}-544.8n+178.8$	$634.5n^{2}-65.55n+222$
$M_{k}RH$	$3240n^{2}-2952n+936$	$236.7n^{2}-238.1n+79.6$	$225n^{2}-211n+68$
$M_{k}RHZ$	$450n^{2}-458n+154$	$10260n^{2}-9708n+3144$	$14580n^{2}-15228n+5184$
$M_{k}RBM$	$2025n^{2}-1899n+612$	$4806n^{2}-4602n+1500$	$6183n^{2}-6429n+2184$
$M_{k}RTM$	$2457n^{2}-2235n+708$	$7722n^{2}-7302n+2364$	$11043n^{2}-11505n+3912$
$M_{k}RGBM$	$161.0424n^{2}-161.2552n+53.7872$	$122.6016n^{2}-122.3568n+40.7448$	$114.9231n^{2}-110.9846n+36.3385$

**TABLE 4:** Modified reverse degree indices of RCF structure for variable parameters k = 1, 2, and 3.

Rectangular coronene fractal structures			
$M_{k}RTI$	k=1	k=2	k=3
$M_{k}RM_{1}$	10m+158n+276mn	12m+276n+504mn	274n−10m+588mn
$M_{k}RM_{2}$	11m+109n+174mn	22m+326n+564mn	331n−31m+786mn
$M_{k}RF$	26m+238n+372mn	48m+672n+1152mn	742n−46m+1668mn
$M_{k}RS$	7.5301m+114.0178n+197.9154mn	8.7654m+196.5618n+358.0622mn	−5.7357m+200.4243n+423.7912mn
$M_{k}RGA$	0.7711m+57.8563n+112.6274mn	93.2982n−2.9404m+198.3579mn	0.4641m+56.3205n+110.7846mn
$M_{k}RReZ_{1}$	98n−2m+204mn	52.333n−0.333 m+106mn	6m+66n+108mn
$M_{k}RReZ_{2}$	2.1667m+37.8333n+67mn	2.8m+68n+124.8mn	63.5n−3.5m+141mn
$M_{k}RH$	47.3333n−1.3333m+100mn	25.8333n−0.2333m+52.6mn	2.3333m+29.6667n+50mn
$M_{k}RHZ$	48m+456n+720mn	92m+1324n+2280mn	1404n−108m+3240mn
$M_{k}RBM$	21m+267n+450mn	34m+602n+1068mn	605n−41m+1374mn
$M_{k}RTM$	37m+347n+546mn	70m+998n+1716mn	1073n−77m+2454mn
$M_{k}RGBM$	17.8236n−0.0353m+35.7882mn	0.0406m+13.7037n+27.2443mn	0.6564m+14.0821n+25.5385mn

## 4 Evaluation of graph entropy

A mathematical foundation for assessing a system’s randomness and uncertainty is provided by Shannon’s concept of entropy, which quantifies the content of possibility distributions. For a discrete random variable $\left(x_{1},x_{2},\ldots ,x_{n}\right)$ with chances $\left(h\left(x_{1}\right),h\left(x_{2}\right),\ldots ,h\left(x_{n}\right)\right)$ , thus Shannon’s entropy (H) , is expressed as:

$$H=-\overset{n}{\underset{i=1}{\sum }}h\left(x_{i}\right)\log_{2}h\left(x_{i}\right),$$

where $h\left(x_{i}\right)=\frac{N_{i}}{N}$ , $N_{i}$ represents the frequency of a specific outcome $x_{i}$ , and N is the total number of outcomes (Shannon, 1948). The information obtained from measuring the system is captured by using the logarithm base-2 to validate the entropy values in bits. This equation has a significant analogy to thermodynamic entropy, which quantifies the randomness of states in a physical system (Mowshowitz and Dehmer, 2012; Sabirov and O-sawa, 2015). In Physics, thermodynamic entropy is used to assess microstates, while Shannon’s entropy is generally applicable to abstract systems, such as graphs, and allows for the analysis of their structural complexity using attributes like vertices and edges (Mowshowitz, 1968).

Based on this foundation, incorporating topological indices into the entropy framework appears as a robust approach to assess molecular complexity. This approach focuses on graph edges and uses topological indices (TIs) , which are mathematical structural characterization of molecular graphs (Arockiaraj et al., 2023d). The probability given to each edge of a molecular graph G , with edges ab∈E(G) is defined as $\frac{f\left(ab\right)}{M_{k}RTI}$ , where f(ab) is a modified reverse degree-based function and $M_{k}RTI$ is the associated index. The graph entropy is expressed as:

$$I_{M_{k}RTI}=-\underset{ab\in E\left(G\right)}{\sum }\frac{f\left(ab\right)}{M_{k}RTI}\log_{2}\left(\frac{f\left(ab\right)}{M_{k}RTI}\right).$$

Further the graph entropy equation simplifies as:

$$I_{M_{k}RTI}=\log_{2}\left(M_{k}RTI\left(G\right)\right)-\frac{1}{M_{k}RTI\left(G\right)}\underset{ab\in E\left(G\right)}{\sum }f\left(ab\right)\log_{2}f\left(ab\right).$$

By employing specific topological indices, the simplified representation makes it easier to calculate graph entropy for a molecular graphs of coronene fractals. For example, the modified first Zagreb index applied to a ZHCF (n) structure. The result of substituting into the entropy formula is:

$$I_{M_{k}RM_{1}}=\log_{2}\left(M_{k}RM_{1}\right)-\frac{1}{M_{k}RM_{1}}\underset{ab\in E\left(G\right)}{\sum }f\left(ab\right)\log_{2}f\left(ab\right).$$

Employing degree-based edge partitions presented in Table 1, the entropy of ZHCF (n) when k=1 and $M_{1}RM_{1}$ is:

$$\begin{array}{lll} I_{M_{1}RM_{1}}\left(\text{ZHCF}\left(n\right)\right) & =\log_{2}\left(414n^{2}+30n\right)-\frac{1}{414n^{2}+30n}\left[18n^{2}+6n\times \left(4\times \log_{2}\left(4\right)\right)\right.\\ & \left.\;+36n^{2}+12n\times \left(3\times \log_{2}\left(3\right)\right)+\left(117n^{2}-15n\right)\times \left(2\times \log_{2}\left(2\right)\right)\right]\text{For }n=2,\text{ we obtain:}I_{M_{1}RM_{1}}\left(\text{ZHCF}\left(2\right)\right) & =\log_{2}\left(1716\right)-\frac{1}{1716}\left[2346.82110036\right]=9.37722247 \end{array}$$

The entropy expressions for all configuration of coronene fractals are too extensive to display. Therefore, Tables 5–7 present the comparison of numerical values of modified reverse degree-based entropy levels for the fractal structures. For rectangular coronene fractals, we assume m=n .

**TABLE 5:** Comparison of entropy levels for ZHCF at k=1 , k=2 , and k=3 .

$I_{M_{k}RTI}$	Zigzag hexagonal coronene fractal structure											
	k=1				k=2				k=3			
	n=2	n=3	n=4	n=5	n=2	n=3	n=4	n=5	n=2	n=3	n=4	n=5
$I_{M_{k}RM_{1}}$	9.3772	10.5436	11.3720	12.0148	9.4136	10.5797	11.4078	12.0505	9.3651	10.5332	11.3624	12.0057
$I_{M_{k}RM_{2}}$	9.2082	10.3750	11.2037	11.8468	9.3605	10.5271	11.3556	11.9986	9.2105	10.3858	11.2186	11.8641
$I_{M_{k}RF}$	9.1938	10.3591	11.1871	11.8297	9.3556	10.5221	11.3505	11.9934	9.2797	10.4518	11.2830	11.9276
$I_{M_{k}RS}$	9.3734	10.5397	11.3680	12.0109	9.4128	10.5788	11.4070	12.0497	9.3724	10.5404	11.3695	12.0127
$I_{M_{k}RGA}$	9.4300	10.5958	11.4237	12.0663	9.4304	10.5961	11.4241	12.0667	9.4278	10.5937	11.4217	12.0643
$I_{M_{k}RReZ_{1}}$	9.3988	10.5656	11.3941	12.0370	9.4181	10.5842	11.4124	12.0551	9.2860	10.4524	11.2808	11.9237
$I_{M_{k}RReZ_{2}}$	9.3825	10.5491	11.3776	12.0205	9.4149	10.5810	11.4091	12.0518	9.3367	10.5058	11.3356	11.9792
$I_{M_{k}RH}$	9.3911	10.5581	11.3867	12.0297	9.4165	10.5827	11.4108	12.0536	9.2991	10.4667	11.2957	11.9390
$I_{M_{k}RHZ}$	9.2027	10.3687	11.1970	11.8399	9.3583	10.5248	11.3533	11.9962	9.2519	10.4253	11.2571	11.9020
$I_{M_{k}RBM}$	9.3240	10.4908	11.3194	11.9624	9.3896	10.5560	11.3843	12.0272	9.2934	10.4646	11.2953	11.9396
$I_{M_{k}RTM}$	9.2001	10.3658	11.1940	11.8368	9.3574	10.5240	11.3524	11.9953	9.2625	10.4353	11.2669	11.9117
$I_{M_{k}RGBM}$	9.4230	10.5890	11.4171	12.0597	9.4262	10.5920	11.4201	12.0627	9.4056	10.5718	11.4001	12.0428

**TABLE 6:** Comparison of entropy levels for AHCF at k=1 , k=2 , and k=3 .

$I_{M_{k}RTI}$	Armchair hexagonal coronene fractal structures											
	k=1				k=2				k=3			
	n=2	n=3	n=4	n=5	n=2	n=3	n=4	n=5	n=2	n=3	n=4	n=5
$I_{M_{k}RM_{1}}$	10.1830	11.6202	12.5802	13.3006	10.2193	11.6560	12.6158	13.3362	10.1716	11.6105	12.5712	13.2921
$I_{M_{k}RM_{2}}$	10.0142	11.4519	12.4122	13.1329	10.1664	11.6038	12.5640	13.2845	10.0201	11.4661	12.4301	13.1528
$I_{M_{k}RF}$	9.9991	11.4353	12.3951	13.1154	10.1615	11.5987	12.5588	13.2793	10.0880	11.5308	12.4933	13.2152
$I_{M_{k}RS}$	10.1792	11.6162	12.5762	13.2967	10.2185	11.6552	12.6150	13.3353	10.1789	11.6175	12.5782	13.2990
$I_{M_{k}RGA}$	10.2356	11.6720	12.6317	13.3519	10.2360	11.6724	12.6320	13.3523	10.2334	11.6699	12.6296	13.3499
$I_{M_{k}RReZ_{1}}$	10.2048	11.6422	12.6024	13.3229	10.2238	11.6606	12.6204	13.3408	10.0918	11.5290	12.4891	13.2096
$I_{M_{k}RReZ_{2}}$	10.1885	11.6258	12.5859	13.3063	10.2206	11.6573	12.6172	13.3375	10.1437	11.5835	12.5448	13.2659
$I_{M_{k}RH}$	10.1972	11.6348	12.5951	13.3157	10.2222	11.6591	12.6189	13.3393	10.1054	11.5438	12.5045	13.2253
$I_{M_{k}RHZ}$	10.0083	11.4452	12.4053	13.1258	10.1642	11.6015	12.5616	13.2821	10.0607	11.5047	12.4678	13.1900
$I_{M_{k}RBM}$	10.1300	11.5676	12.5279	13.2484	10.1955	11.6326	12.5925	13.3130	10.1012	11.5431	12.5053	13.2269
$I_{M_{k}RTM}$	10.0056	11.4422	12.4022	13.1226	10.1633	11.6006	12.5607	13.2812	10.0711	11.5146	12.4775	13.1995
$I_{M_{k}RGBM}$	10.2287	11.6653	12.6251	13.3453	10.2318	11.6683	12.6280	13.3483	10.2114	11.6483	12.6082	13.3286

**TABLE 7:** Comparison of entropy levels for RCF where (m=n) at k=1 , k=2 , and k=3 .

$I_{M_{k}RTI}$	Rectangular coronene fractal structure											
	k=1				k=2				k=3			
	n=2	n=3	n=4	n=5	n=2	n=3	n=4	n=5	n=2	n=3	n=4	n=5
$I_{M_{k}RM_{1}}$	9.1164	10.1830	10.9586	11.5688	9.1529	10.2193	10.9947	11.6047	9.1031	10.1716	10.9482	11.5590
$I_{M_{k}RM_{2}}$	8.9473	10.0142	10.7901	11.4005	9.0995	10.1664	10.9422	11.5524	8.9443	10.0201	10.8008	11.4143
$I_{M_{k}RF}$	8.9337	9.9991	10.7741	11.3840	9.0947	10.1615	10.9371	11.5474	9.0154	10.0880	10.8669	11.4791
$I_{M_{k}RS}$	9.1126	10.1792	10.9548	11.5649	9.1521	10.2185	10.9939	11.6039	9.1106	10.1789	10.9554	11.5661
$I_{M_{k}RGA}$	9.1695	10.2356	11.0108	11.6207	9.1699	10.2360	11.0112	11.6211	9.1673	10.2334	11.0087	11.6186
$I_{M_{k}RReZ_{1}}$	9.1376	10.2048	10.9806	11.5909	9.1574	10.2238	10.9992	11.6093	9.0253	10.0918	10.8674	11.4776
$I_{M_{k}RReZ_{2}}$	9.1216	10.1885	10.9642	11.5744	9.1542	10.2206	10.9960	11.6060	9.0742	10.1437	10.9209	11.5320
$I_{M_{k}RH}$	9.1298	10.1972	10.9731	11.5835	9.1558	10.2222	10.9977	11.6078	9.0376	10.1054	10.8818	11.4924
$I_{M_{k}RHZ}$	8.9422	10.0083	10.7838	11.3939	9.0973	10.1642	10.9399	11.5501	8.9869	10.0607	10.8403	11.4530
$I_{M_{k}RBM}$	9.0630	10.1300	10.9059	11.5162	9.1288	10.1955	10.9711	11.5812	9.0296	10.1012	10.8796	11.4915
$I_{M_{k}RTM}$	8.9397	10.0056	10.7809	11.3909	9.0965	10.1633	10.9390	11.5492	8.9977	10.0711	10.8504	11.4629
$I_{M_{k}RGBM}$	9.1624	10.2287	11.0040	11.6140	9.1656	10.2318	11.0071	11.6170	9.1448	10.2114	10.9869	11.5970

The entropy stages provided in Tables 5–7 monitor dynamic variation throughout the three configurations of coronene fractals for k=1,2 , and 3. Notably, entropy values continually peaks at k=2 in comparison to k=1 and k=3 . The entropy measures differ slightly in their decimal values across all indices. Among the configurations, AHCF demonstrates slightly higher entropy values than the other coronene structures, while RCF exhibits lower entropy values, indicating greater structural stability. However, direct comparisons of complexity measures across these fractal structure are complicated by differences in the number of edges. We utilize relative measures, including structural information content (SIC) and bond information content (BIC), derived from the computed entropy values. These metrics provide a exact evaluation of the structural complexity and stability of the three coronene fractal configurations.

### 4.1 Relative complexity metrics

This subsection offers numerical and graphical estimation of complexity across the configurations of coronene fractals, emphasizing the importance of accounting for molecular size differences. Since graph entropy values are depending on the size of the molecular graph, the application of relative complexity measures has become essential for higher comparisons among molecular systems of varying dimensions (Dehmer, 2008). To address this, two normalized measures, namely structural information content (SIC) and bond information content (BIC), are introduced. Graph entropy alone may not adequately reflect structural complexity, especially for systems with differing dimensional sizes, highlighting the necessity of employing relative metrics (Bonchev and Trinajstić, 1982; Sabirov and Shepelevich, 2021). The maximum entropy concept is used to establish these metrics, where the limiting entropy value for $I_{M_{k}RTI}$ is defined as $I_{M_{k}RTI}^{\text{max}}=\log_{2}\left(M_{k}RTI\right)$ (Junias et al., 2024). This leads to SIC, which quantifies molecular structure and the most useful information, as shown below:

$${\text{SIC}}_{M_{k}RTI}=\frac{I_{M_{k}RTI}}{I_{M_{k}RTI}^{\text{max}}}.$$ (13)

Similarly, BIC includes a molecular graph where edges are counted to compute relative complexity. The formula for the BIC normalizes the entropy using the logarithmic scale of the total number of edges, as shown here:

$${\text{BIC}}_{M_{k}RTI}=\frac{I_{M_{k}RTI}}{\log_{2}|E\left(G\right)|}.$$ (14)

From Equations 13, 14, we calculate the SIC and BIC measures for coronene fractals. The analysis focuses on the entropy values of the Zagreb index when k=2 , where $I_{M_{k}RTI}=I_{M_{2}RM_{1}}$ providing insight into the relative complexity assessment between the fractals. For example the ZHCF(3) system with |E(G)|=1548 , the Zagreb index value $I_{M_{k}RTI}=I_{M_{2}RM_{1}}=10.57965167$ and $I_{M_{k}RTI}^{\text{max}}=\log_{2}\left(M_{2}RM_{1}\right)=\log_{2}\left(6912\right)$ . The values calculated by equations for SIC and BIC are as follows.

$${\text{SIC}}_{M_{2}RM_{1}}=\frac{I_{M_{2}RM_{1}}}{\log_{2}\left(M_{2}RM_{1}\right)}=\frac{10.57965167}{12.7548875}=0.829458642$$

$${\text{BIC}}_{M_{2}RM_{1}}=\frac{I_{M_{2}RM_{1}}}{\log_{2}|E\left(G\right)|}=\frac{10.57965167}{10.59618976}=0.998439242$$

**FIGURE 3:**
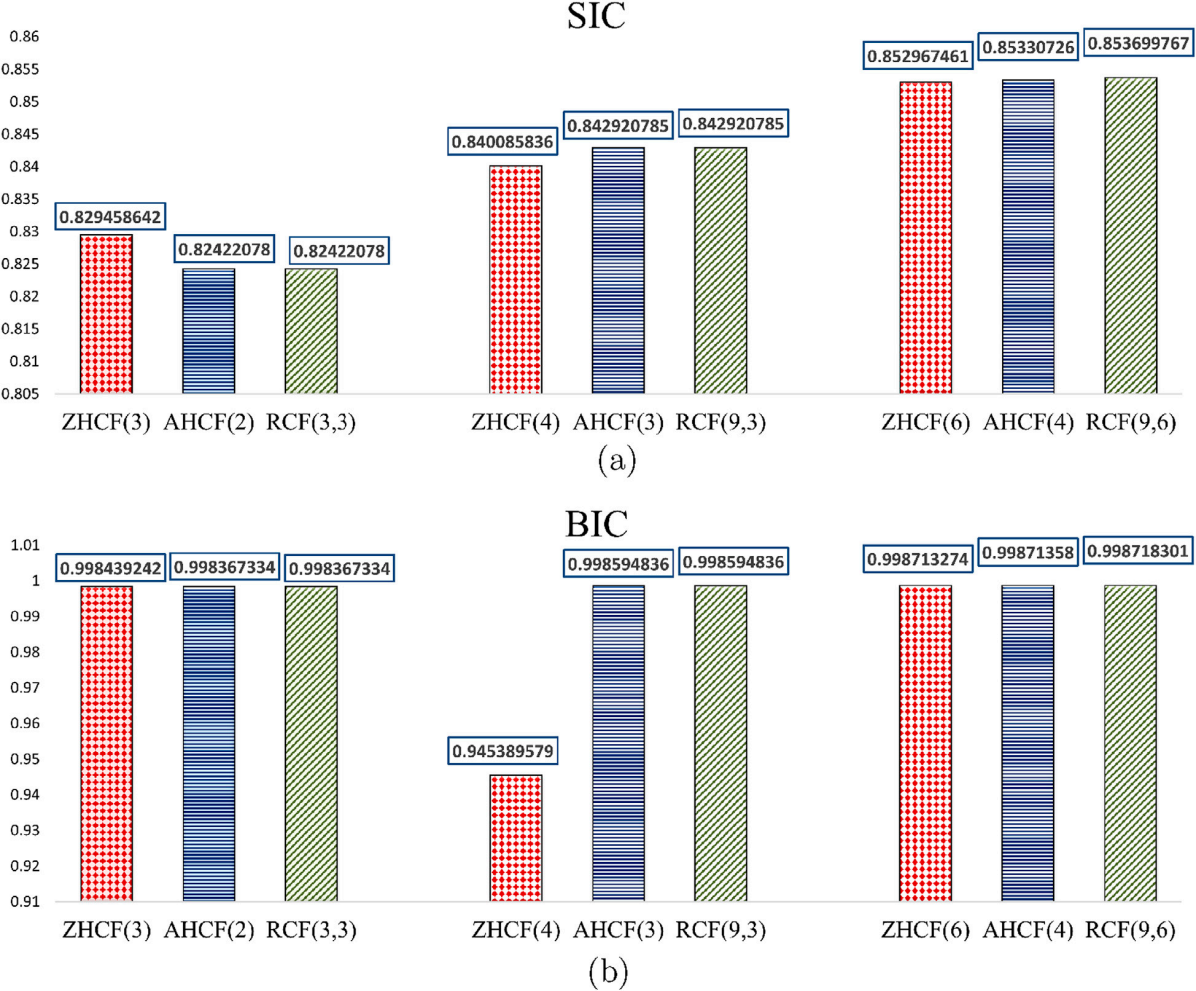
Graphical comparison of complexity measures among zigzag, armchair and rectangular coronene fractals. **(a)** Comparison of SIC measures across the range of *k* values for coronene fractals. **(b)** Comparison of BIC measures across the range of *k* values for coronene fractals.

The SIC and BIC measures for other coronene fractals across various vertex ranges, are presented in Table 8. These relative complexity measures offer a comparative analysis of complexity across different sizes, with values ranging from 0 to 1, where 1 indicates the highest complexity and 0 the lowest. The SIC and BIC measures, are shown in Table 8, with a graphical comparison in Figure 3.

**TABLE 8:** Relative complexity measures of three classes of coronene structures.

Vertex ranges	Structures	$I_{M_{2}RM_{1}}$	$I_{M_{2}RM_{1}}^{\text{max}}$	$\log_{2}|E\left(G\right)|$	SIC	BIC
900–1152	ZHCF(3)	10.57965	12.75489	10.59619	0.829459	0.998439
	AHCF(2)	10.2193	12.39874	10.23601	0.824221	0.998367
	RCF(3,3)	10.2193	12.39874	10.23601	0.824221	0.998367
2040–2424	ZHCF(4)	11.40779	13.57932	12.06676	0.840086	0.94539
	AHCF(3)	11.65602	13.82814	11.67243	0.842921	0.998595
	RCF(9,3)	11.65602	13.82814	11.67243	0.842921	0.998595
4572–4872	ZHCF(6)	12.57579	14.74357	12.59199	0.852967	0.998713
	AHCF(4)	12.61584	14.78463	12.63209	0.853307	0.998714
	RCF(9,6)	12.6542	14.82277	12.67043	0.853700	0.998718

Table 8; Figure 3 show that RCF and AHCF exhibit similar complexity values at small scales. However, with increasing size, the rectangular fractals exhibits slightly higher complexity compared to armchair configuration, while the zigzag-based coronene exhibits lower complexity than all other configurations because BIC is evaluated based on number of bonds in molecular graph and SIC obtained from maximum entropy. These two analyzes facilitate better comparisons, and help to determine the most appropriate indicator of complexity measures for molecular system.

From Figure 3; Tables 5–8, greater entropy variations are observed among the three configurations for smaller structures, while for the largest structure, all configurations approach the 2D graphitic sheet, and their entropy values converge to a limit. However, two types of isentropic structures exist: AHCF(2) and RCF(3,3) have the same number of vertices (900) and edges (1206); similarly, AHCF(3) and RCF(9,3) share the same number of vertices (2424) and edges (3264). Thus, we use spectral properties for a more conclusive analysis of stability.

## 5 Analysis of spectral properties in coronene fractals

This section focuses on the spectral properties of coronene fractals, using metrics derived from their graph spectra. Since these structures are two-dimensional and satisfy the Coulson-Rushbrook theorem, this method is more effective for analysis. It is not practical to perform complete Abinitio calculations for complex systems such as AHCF(3) and RCF(9,3) with 2424 vertices and spectral eigenvalues (Arockiaraj et al., 2022). Consequently, machine learning techniques are needed to efficiently estimate stability in large, fractal structures. Significant spectal and energy properties such as total π -electron energy, spectral diameter, HOMO-LUMO energy gap, delocalization energy, and resonance energy, are determined by combinatorial analysis of graph spectra (Prabhu et al., 2024). These parameters provide valuable insights into the thermodynamic and kinetic stability of the coronene fractals under investigation.

The total π -electron energy $\left(E_{\pi }\right)$ is a critical measure of electronic stability in conjugated systems (Gutman and Trinajstić, 1972; Gutman, 1978). For coronene fractals, including zigzag, armchair, and rectangular patterns, $E_{\pi }$ is calculated using the eigenvalues $\left(\lambda_{i}\right)$ of the graph spectra of the molecular graph (Kalaam et al., 2024; Graovac et al., 1977). For a system with p atoms:

$$E_{\pi }=\left\{\begin{array}{cc} 2\overset{p/2}{\underset{i=1}{\sum }}\lambda_{i},\; & \text{if }p\text{ is even},\\ \lambda_{\left(p+1\right)/2}+2\overset{\left(p-1\right)/2}{\underset{i=1}{\sum }}\lambda_{i},\; & \text{if }p\text{ is odd}. \end{array}\right.$$

The π -electron distribution depends on whether p is even or odd.

The HOMO-LUMO energy gaps, defined as the difference between the highest molecular orbital (HOMO) denoted $\lambda_{H}$ and the lowest unoccupied molecular orbital (LUMO) denoted $\lambda_{L}$ . It plays an important role in analyzing molecular reactivity and kinetic stability. These difference is calculated by subtracting the lowest positive eigenvalue from the highest negative eigenvalue from the graph spectrum, expressed as $\Delta_{G}=\lambda_{H}-\lambda_{L}$ (Wu et al., 2018; Li et al., 2013). Larger HOMO-LUMO energy differences indicate increased kinetic stability and low chemical reactivity, as more energy is required to transfer an electron from HOMO to LUMO, thus decreasing the chemical reactivity however this difference does not directly reflect thermodynamic stability.

Thermodynamic stability is closely related to parameters such as delocalization and resonance energies, which generally increase with molecule size, increasing the stability. The delocalization energy ${\left(E_{\text{Deloc}}\right)}_{\text{per bond}}$ , is calculated as ${\left(E_{\text{Deloc}}\right)}_{\text{per bond}}=E_{\pi }-|V\left(G\right)|$ . Kekul $\overset{´}{e}$ counts (KC), which reflect the number of Kekul $\overset{´}{e}$ resonance structures in coronene fractals, are used to compute resonance energies as coronene fractals are benzenoid systems and bipartite graphs. Thus (KC) is derived from the square root of the constant term of the characteristic polynomial (Balasubramanian, 2023). According to Herndon’s definition of resonance, $RE_{\text{per bond}}=\frac{1}{\left|V\left(G\right)\right|}\left.\left(1.185\times \ln\left(\text{KC}\right)\right)\right)$ (Herndon and Ellzey, 1974). The increase in size of the coronene fractals increases both delocalization and resonance energies. Because of the stabilization of the molecular orbitals (Mazouin et al., 2022), the HOMO-LUMO energy gap decreases with increasing molecular size. The spectral diameter SD is calculated as the difference between the maximum and minimum eigenvalues: $SD=\lambda_{max}-\lambda_{min}$ . These graph spectra based energy properties were assessed using programs such as newGRAPH and MATLAB software (Stevanović et al., 2021; MATLAB, 2022). Table 9 displays the results, which are given in β units.

**TABLE 9:** Energetic properties of three classes of polycyclic aromatic hydrocarbons.

Structure	$E_{\pi }$	$\left(\Delta_{G}\right)$ gaps	${\left(E_{\pi }\right)}_{\text{per bond}}$	${\left(E_{\text{Deloc}}\right)}_{\text{per bond}}$	${\left(RE\right)}_{\text{per bond}}$	SD
ZHCF(1)	194.662 β	0.7638 β	1.475 β	0.474712 β	0.156614 β	5.5937 β
ZHCF(2)	765.926 β	0.65732 β	1.484 β	0.484353 β	0.159251 β	5.64926 β
ZHCF(3)	1713.792 β	0.62768 β	1.488 β	0.487666 β	0.160157 β	5.65958 β
ZHCF(4)	3038.259 β	0.61518 β	1.489 β	0.489343 β	0.160615 β	5.66336 β
AHCF(1)	194.662 β	0.7638 β	1.475 β	0.474712 β	0.156614 β	5.5937 β
AHCF(2)	1337.189 β	0.63774 β	1.486 β	0.485766 β	0.159636 β	5.65682 β
AHCF(3)	3609.522 β	0.61322 β	1.489 β	0.489077 β	0.160541 β	5.66408 β
RCF(1)	194.662 β	0.7638 β	1.475 β	0.474712 β	0.156614 β	5.5937 β
RCF(2)	640.3919 β	0.66974 β	1.482 β	0.482389 β	0.158714 β	5.64364 β
RCF(3)	1337.19 β	0.6379 β	1.486 β	0.485766 β	0.159637 β	5.65572 β
RCF(4)	2285.055 β	0.6229 β	1.488 β	0.487666 β	0.160156 β	5.66072 β

**FIGURE 4:**
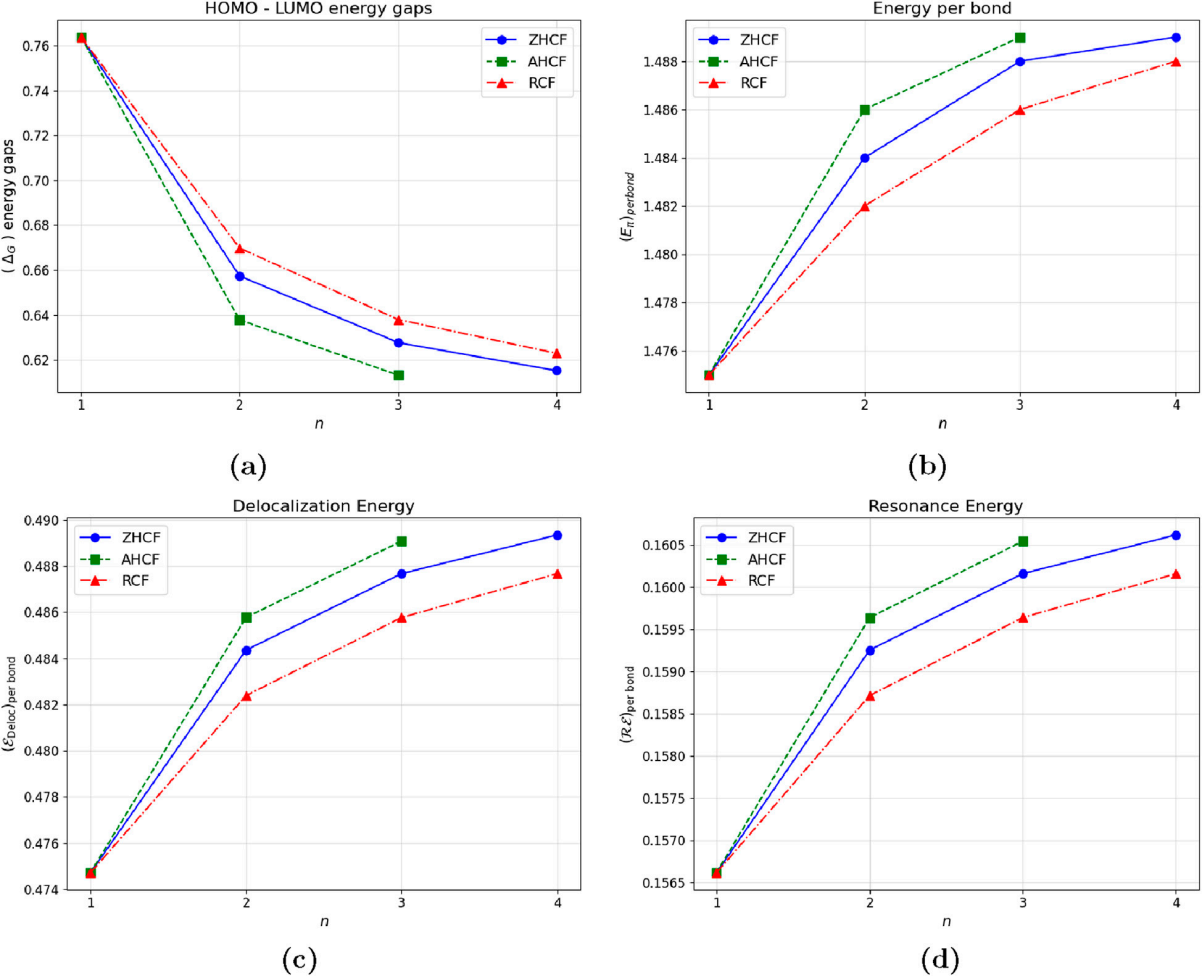
Graphical representation of energetic properties across kekulene tessellations. **(a)** HOMO-LUMO energy gap **(b)** Energy per bond **(c)** Delocalization energy per bond **(d)** Resonance energy per bond.

The data present in Table 9; Figure 4 show that the HOMO-LUMO energy gaps decrease as the size of coronene structures increase. This suggests that larger structures have more electronic delocalization and resonance energy, which results in lower energy differences between the highest occupied molecular orbital (HOMO) and the lowest unoccupied molecular orbital (LUMO). Meanwhile, both delocalization and resonance energy show an increasing trend, reflecting enhanced stability and conjugation within these structures. Among the fractal configurations analyzed, the rectangular coronene fractals have the largest HOMO-LUMO energy gaps, suggesting high kinetic stability, lower reactivity, and the lowest delocalization and resonance energies. On the other hand, armchair coronene fractals display the smallest HOMO-LUMO energy gaps, indicating less kinetic stability, larger chemical reactivity, higher electron delocalization, and resonance energies, all of which lead to greater stability with efficient electron transfer. This study emphasizes the significance of structural configuration on stability and reactivity in coronene fractals.

## 6 Predictive models

The prediction of the spectral properties of chemical structure by graph-entropy measures utilizes structure-property models which play an important role in characterizing and prediction chemical properties using topological indices (Raza et al., 2024; Rauf et al., 2022) These models offer a cost-effective alternative to experimental studies, offering reliability, accuracy and robustness (Hayat et al., 2019). For coronene fractals, we examine the relationship between spectral features and entropy measurements obtained from the reverse degree-based indices. Our findings show that there is a better correlation between the entropy measures and spectral properties, except for the HOMO-LUMO energy gap, which exhibits negative correlation due to its decrease in energy gaps with increasing system size. As noted in the previous section, the first Zagreb index was employed to compare relative complexity measures among the structures. We found that entropy measures associated with $M_{2}RM_{1}$ demonstrate the strongest correlation with spectral characteristics. Linear regression analysis was used to develop predictive models for spectral properties. The linear regression equation given as $P=R\cdot \left(I_{M_{k}RTI}\right)+c$ , where P is the spectral properties, R is the regression coefficient, and c is the regression constant. The statistical parameters such as $r^{2}$ , $r^{2}$ , F -values, and S.E are utilized to validate model’s performance.

**FIGURE 5:**
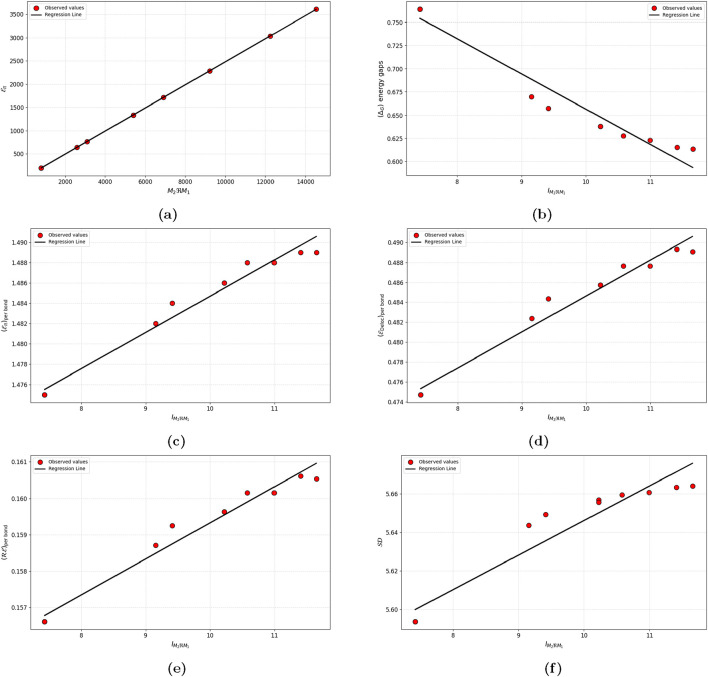
Linear regression models for the energetic properties. **(a)** Total π electron energy **(b)** Homo-Lumo energy gap **(c)** Energy per bond **(d)** Delocalization energy **(e)** Resonance energy per bond **(f)** Spectral diameter.

The regression models optimized to predict spectral characteristics are given detailed in Table 10 and illustrated in Figure 5. The selection was based on their unique performance indicators, such as $r^{2}$ , adjusted $r^{2}$ , high F -values, in addition to reduced error (SE) objectives. These metrics confirm the reliability and accuracy of the models. The developed models are particularly effective in estimating the energy value of high-aspect ratio coronene explosions. An efficient method based on linear regression was used to ensure accurate predictions while minimizing computational complexity.

**TABLE 10:** Statistically derived optimal regression models for predicting energetic properties.

P	Optimal regression equation	$r^{2}$	$adj\left(r^{2}\right)$	SE	F
$E_{\pi }$	0.248 $\left(M_{2}RM_{1}\right)$ –2.775	0.999	0.998	0.886	17582849.51
$\left(\Delta_{G}\right)$ energy gaps	−0.038 $\left(I_{M_{2}RM_{1}}\right)$ +1.036	0.951	0.946	0.015	175.129
${\left(E_{\pi }\right)}_{\text{per bond}}$	0.004 $\left(I_{M_{2}RM_{1}}\right)$ +1.448	0.973	0.970	0.001	329.292
${\left(E_{\text{Deloc}}\right)}_{\text{per bond}}$	0.004 $\left(I_{M_{2}RM_{1}}\right)$ +0.448	0.973	0.970	0.001	329.292
${\left(RE\right)}_{\text{per bond}}$	0.001 $\left(I_{M_{2}RM_{1}}\right)$ +0.149	0.973	0.970	0.006	327.889
SD	0.018 $\left(I_{M_{2}RM_{1}}\right)$ +5.467	0.915	0.906	0.009	96.845

## 7 Conclusion

In this paper, we develop topological expressions based on modified reverse degree-based indices for three configurations of two-dimensional coronene fractals. These indices capture structural complexities and are effective in predicting physico-chemical properties. The computed indices function as graph-based metrics for evaluating entropy levels and relative complexity. The resulting entropy values offer insights into the structural challenges of these fractal systems, providing a foundation for further investigation into their properties. When paired with graph spectra, these approaches form a comprehensive machine learning framework for efficiently and accurately computing the spectral and thermodynamic properties of fractals and other two-dimensional materials. By integrating graph-theoretic methods with advanced statistical techniques, this study contributes to the development of improved computational chemistry algorithms, particularly for QSAR and QSPR studies aimed at predicting the stability and characteristics of complex chemical systems.

## Data Availability

The original contributions presented in the study are included in the article/supplementary material, further inquiries can be directed to the corresponding author.
